# The TacTip Family: Soft Optical Tactile Sensors with 3D-Printed Biomimetic Morphologies

**DOI:** 10.1089/soro.2017.0052

**Published:** 2018-04-01

**Authors:** Benjamin Ward-Cherrier, Nicholas Pestell, Luke Cramphorn, Benjamin Winstone, Maria Elena Giannaccini, Jonathan Rossiter, Nathan F. Lepora

**Affiliations:** ^1^ Department of Engineering Mathematics, University of Bristol, Bristol, United Kingdom.; ^2^ Bristol Robotics Laboratory, Bristol, United Kingdom.

**Keywords:** tactile sensors, dexterous manipulation, soft sensors

## Abstract

Tactile sensing is an essential component in human–robot interaction and object manipulation. Soft sensors allow for safe interaction and improved gripping performance. Here we present the TacTip family of sensors: a range of soft optical tactile sensors with various morphologies fabricated through dual-material 3D printing. All of these sensors are inspired by the same biomimetic design principle: transducing deformation of the sensing surface via movement of pins analogous to the function of intermediate ridges within the human fingertip. The performance of the TacTip, TacTip-GR2, TacTip-M2, and TacCylinder sensors is here evaluated and shown to attain submillimeter accuracy on a rolling cylinder task, representing greater than 10-fold super-resolved acuity. A version of the TacTip sensor has also been open-sourced, enabling other laboratories to adopt it as a platform for tactile sensing and manipulation research. These sensors are suitable for real-world applications in tactile perception, exploration, and manipulation, and will enable further research and innovation in the field of soft tactile sensing.

## Introduction

The sense of touch is essential for interacting physically with our environment,^1^ such as with other humans in social interactions.^2^ In robotics, tactile feedback is essential for complex precision manipulation tasks^3^ as well as for safe human–robot interaction. Developing robust, customizable tactile sensors is thus an important task that could drive advances in the safety, interactivity, and manipulation capabilities of robots.

A large variety of tactile sensors have been developed over the years,^4^ relying on various technologies such as capacitive taxels, resistive wires, and piezoelectric materials. However, there is a general lack of cheap customizable tactile sensors in the field, which is hampering the ease of researching applications of robot touch. We aim to develop these types of tactile sensors and make them available through open resources such as the soft robotics toolkit.^5^

Our sensors are soft, with a compliant modular tip that protects sensitive electronic parts from physical contact with objects. When integrated into robotic grippers, compliant sensors have also been shown to improve grasping.^6^ Developing soft sensors is also key for safe and comfortable human–robot interaction.^3^

Advances in multimaterial 3D printing allow researchers to manufacture rapidly prototyped robot hands and sensors with integrated soft surfaces for compliant, adaptable, and sensorized manipulation. Recent work in the application of soft, 3D-printed tactile sensors, for instance, in demonstrating tactile super-resolution,^7^ extends the scope of these devices from prototypes to useful, working versions of sensors whose materials and morphologies can be quickly, easily, and cheaply adapted to suit different practical applications.

**FIG. 1.**
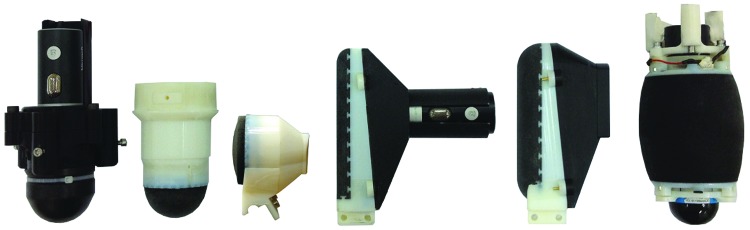
TacTip project sensors. From *left* to *right*: open-TacTip (original), TacTip (improved), TacTip-GR2, TacTip-M2 (flat), TacTip-M2 (round), and TacCylinder.

**FIG. 2.**
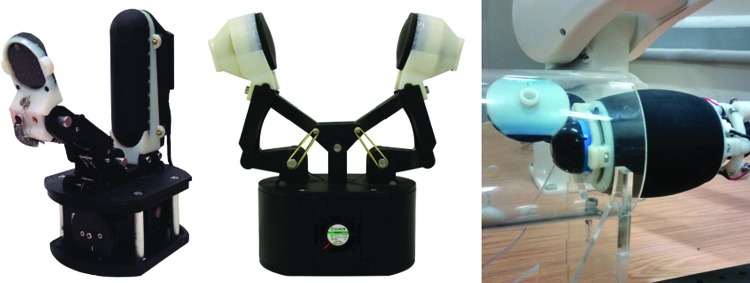
The TacTip-M2 integrated into the open-hand model M2 gripper (*left*), the TacTip-GR2 mounted on the GR2 gripper (*middle*), and the TacCylinder in a simulated tumor detection experiment (*right*).

The aim of this study is to present a suite of soft sensors of various morphologies using 3D printing, including the tactile fingertip (TacTip) by successive modifications of the original cast tip version (Fig. 1). The TacTip^8^ is a low-cost, robust, 3D-printed optical tactile sensor based on a human fingertip and developed at Bristol Robotics Laboratory (BRL). We also introduce the TacTip-M2, TacTip-GR2, and TacCylinder (Fig. 2), more skin-like derived sensors whose fabrication is made possible by multimaterial 3D printing and which are designed for integration in two-fingered grippers and for capsule endoscopy, respectively.

We have open-sourced the open TacTip (www.softroboticstoolkit.com/tactip) to offer other research institutions the opportunity to adopt and adapt our sensor designs, such as to create different morphologies, as we have done with the TacCylinder.^9^ The sensor design can also be modified to integrate with robot hands, as demonstrated with the TacTip-M2 (formerly TacThumb)^10^ and TacTip-GR2.

This article presents the first comparative study of our suite of soft sensors, all of which are highly accurate, being able to localize objects to submillimeter accuracy that demonstrates super-resolved acuity. This high performance of the TacTip family of sensors suggests that analogous designs could result in a range of novel soft complex tactile sensors from regions of tactile skin to tactile feet and proboscises.

## Background

### Related technologies

Most tactile sensors are soft, comprising at least some compliant elements, and rely on a variety of underlying technologies (e.g., strain-gauge,^11^ barometric,^12^ capacitive,^13^ piezoresistive,^14^ piezoelectric^15^ …) to transmit and record tactile information. Here we review existing compliant optical tactile sensors, which relate most closely to our family of TacTip sensors.

At first glance, the design of the Optoforce force/torque sensor (www.optoforce.com) seems closely related to the TacTip, based on its overall shape and design. However, the Optoforce is designed to compute only the overall force and torque of the contact, rather than relaying tactile information that comprises an array of sensor readings across a sensing surface. Force/torque sensing is unlike human touch and inadequate for tasks requiring more information than a force vector, such as multicontact sensing.

An early precursor to the TacTip used a molded transparent dome with a black dotted pattern.^16^ However, the use of ambient light for imaging the dots has drawbacks, including lack of contrast on objects that obscure the ambient lighting and difficulties for automated tracking of the dots; moreover, the design presents difficulties for image recognition of tactile elements independent of lighting conditions.

A further tactile sensor based on optics is the GelSight sensor,^17^ which uses colored lights and photometric stereo to reconstruct highly accurate deformations of its surface. This sensor obtains high resolutions, uses inexpensive materials, and can be made into a portable device. While in many ways it represents an excellent, low-cost optical tactile sensor, it presently requires a flat surface and there would be challenges in adapting it to more complex morphologies such as domed fingertips. Similar considerations apply to the GelForce sensor,^18^ where the sensing surface is a flat elastomer pad.

Other examples include tactile sensors that use an optical waveguide approach,^19–21^ or patterns of dots^22^ or lines^23^ drawn on the inside surface of a flexible skin, tracked by a CCD camera. Some sensors also make use of fiber optics to relay light intensities to the camera,^24^ allowing the sensor's contact area to be made very small, ideal for medical applications.

All tactile sensors have their pros and cons, and ultimately, the best choice will depend on the application. That being said, an important distinction between the TacTip and the sensors described above is the presence of physical pins attached to the sensing surface; these structures mimic intermediate ridges within the human fingertip, giving a biomimetic basis for the sensor design, as described below.

### Inspiration

**FIG. 3.**
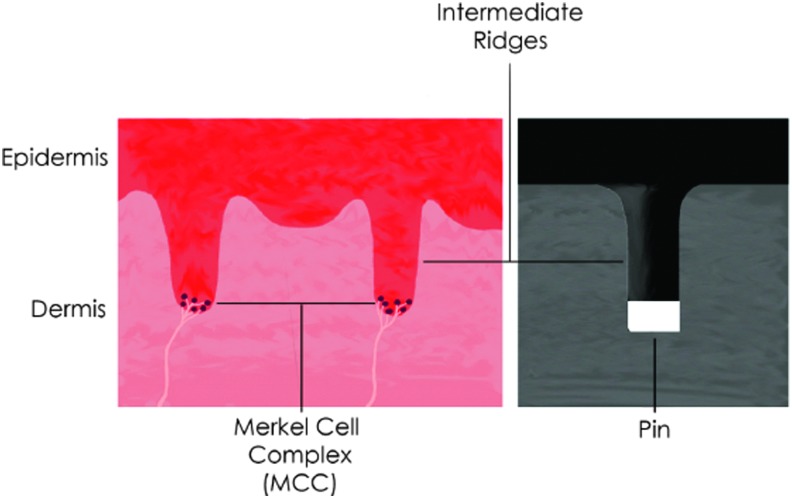
Intermediate ridges in the human skin (*left*) and a corresponding pin in the TacTip (*right*).

**FIG. 4.**
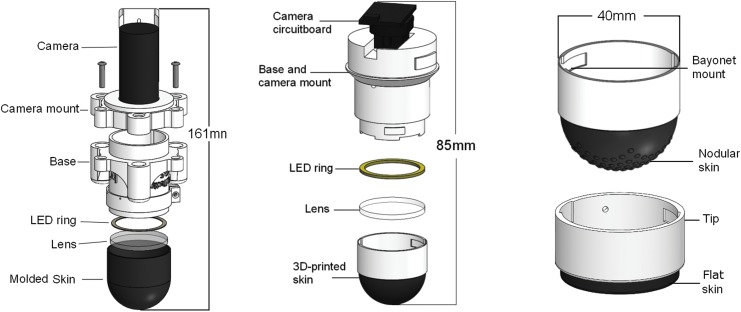
Open-TacTip (*left*): the original version of the sensor comprises a 3D-printed camera mount and base and a cast silicone skin. Improved TacTip (*center*): the redesigned base houses a reassembled webcam, and modular tips with 3D-printed rubber skin. Modular tips (*right*): separate modular tips with a nodular fingerprint (*above*) and flat tip (*below*).

When the human finger makes contact with an object or surface, deformation occurs in the epidermal layers of the skin and the change is detected and relayed by its mechanoreceptors.^1^ Chorley *et al.*^8^ were inspired to consider the behavior of the human glabrous (hairless) skin, as found on the palms of human hands and soles of our feet. They built on previous research showing that the Merkel cell complex of sensory receptors works in tandem with the morphology of the intermediate ridges (Fig. 3) to provide edge encoding of a contacted surface. The TacTip device seeks to replicate this response by substituting intermediate ridges with internal pins on the underside of its soft, skin-like membrane, with optical pin tracking via an internal camera taking the place of mechanosensory transduction of the sensing surface (Fig. 4).

This biomimetic inspiration was recently extended by exploring the role of an artificial fingerprint on tactile sensing with the TacTip.^25^ In that study, an artificial fingerprint consisting of a series of outer nodules on the TacTip's skin was shown to enhance high spatial frequency detection. This finding suggests that the inclusion of artificial fingerprints in biomimetic fingertips will improve their ability to perform tactile tasks such as edge perception, contour following, and fine feature classification, with potential implications for object perception and tactile manipulation.

### Development

The focus of this section is a description of the developments leading to the TacTip sensors presented here (Fig. 4).

The original TacTip^8^ is a soft, robust, and high-sensitivity sensor making use of biomimetic methods for active perception. This sensor has been shown to achieve 40-fold localization super-resolution^7^ and successfully perform tactile manipulation on a cylinder rolling task.^26^

Further efforts to integrate the TacTip for use with 3D-printed robot hands and grippers have led to the development of the TacTip-M2^10^ and TacTip-GR2 sensors, integrated on the M2 gripper^26^ and GR2 gripper,^27^ respectively. These open-source robot hands developed at Yale's GRAB Lab enable the investigation of in-hand tactile manipulation with the TacTip sensors.

The TacCylinder, designed for capsule endoscopy,^28^ is another adaptation of the TacTip design, expanding the range of tasks that can be tackled with TacTip sensors.

The TacTip is thus an extremely useful research tool, due to its low cost, robustness, and adaptability. It fills the current gap in the field for a cheap, compliant, customizable tactile sensor, and can be applied to a number of challenges in industrial robotics, medical robotics, and robotic manipulation.

## Materials and Methods

### Open-TacTip

In the original version of the open-TacTip,^7,8,29^ the base, camera mount, and rigid part of the tip are 3D printed, the skin is cast in VytaFlex 60 silicone rubber, and the pins' tips are (painstakingly) painted white by hand. The tip is filled with optically clear silicone gel (Techsil, RTV27905). In the fabrication of the open-TacTip, emphasis is placed on the low cost of the sensor, straightforward manufacture, and ease of assembly.

To enhance the functionality of the open-TacTip, a series of modifications are made to the original version that was described above. These modifications aim to further reduce cost, minimize the sensor form factor, optimize sensor accuracy, and make the TacTip easier to use and modify. The main modifications to the improved version of the TacTip are summarized in the following section.

### TacTip (improved)

#### 3D-printed skin

Rather than cast the TacTip's skin in silicone rubber, dual-material rapid prototyping with an Objet 3D printer is used to create the sensor's rigid base in hard plastic (Vero White) and its soft skin in a rubber-like material (Tango Black+). This lowers the cost and accelerates the creation of new prototype TacTip skins, by avoiding the time-consuming mold creation and skin casting/painting fabrication stages. In particular, Vero White tips are now printed directly onto the end of the pins avoiding the need to paint them. Examples of different types of skins include tips with a fingerprint^25^ and a rotationally symmetric pin layout.^30^ Three-dimensional printing also increases reproducibility of design. Differences in 3D-printed tip dimensions are based on the accuracy of the 3D printer. Conversely, in molded tips, the skin is molded by hand, introducing variability in skin thickness between tips. While one would expect that 3D-printed skins would be less robust over the long term than their cast counterparts, we have used such 3D-printed skins over months on a daily basis with no obvious drop in performance. Damage is usually due to human error.

The new printing method also provides the opportunity to add complex features to the sensor's skin. For instance, an exterior fingerprint of rubber nodules was included that mechanically coupled to the white pin tips through rigid internal plastic cores. The creation of this complex skin structure was made possible through the use of multimaterial 3D printing and has been shown to improve perceptual acuity at high spatial frequencies.^31^

#### Modularity

To facilitate skin testing and optimization, the skin is printed in a single structure attached to a hard plastic casing (Fig. 4, right panel), forming a tip that connects to the TacTip base with a bayonet mount. The tip (made up of the skin, gel, lens, and plastic casing) is thus a modular component of the sensor, which is easily replaceable or upgradable. By printing tips in this way, it is possible to produce and test different pin layouts and tip structures to optimize the sensor capabilities at a much lower cost than has been possible previously.

**FIG. 5.**
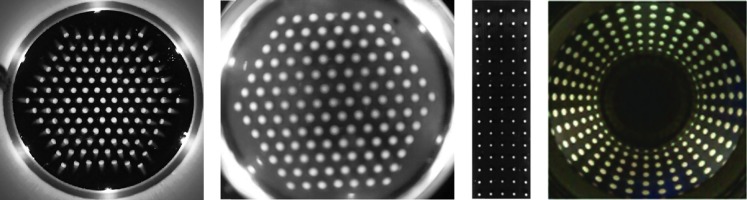
Raw images from the TacTip family of sensors. From *left* to *right*: improved TacTip, TacTip-GR2, TacTip-M2, and TacCylinder. The TacTip images pins with a Microsoft Cinema HD webcam, whereas the TacTip-GR2 uses a Raspberry Pi spycam (Adafruit) and a fisheye lens to reduce the sensor's form factor. The TacCylinder uses a catadioptric mirror to achieve 360° vision.

We are thus able to update the pin layout to a hexagonal projection of 127 pins with a regular spacing when imaged by the camera, an improvement over the uniform geodesic distribution that had been used in past molded TacTips (Fig. 5). This design gives better performance of the pin tracking algorithms during image processing. We also experimented with different skin thicknesses and pin lengths, eventually converging on a 1 mm thick skin with 2.0 mm long pins for the improved TacTip (3° taper) as a good balance between robustness and sensitivity of the pins to deflection. 3D printing was essential in enabling these trial-and-error experiments requiring extensive hardware testing and leading to an improved sensor design.

#### Shorter sensor

A more compact sensor is both easier to deploy in practical scenarios and facilitates integration with a wider range of robot hands and arms. Another important benefit is to reduce the torque on the base if struck laterally, which would be an issue for a long sensor. Thus, we take apart the webcam, retaining only the essential components, and reconnect its circuit boards in a horizontal arrangement (Fig. 4). This shortens the spread along the sensor's long axis, reducing the TacTip's height by approximately a factor of 2 (from 161 to 85 mm). The base and camera mount are also combined into a single piece to simplify the overall sensor design (Fig. 4, middle panel). This sensor design is ideal for mounting as an end-effector to an industrial robot arm, which has been the preferred platform for investigating tactile perception and control in our laboratory.^7,31–33^

#### TacTip-GR2

A version of the TacTip created for integration onto the GR2 gripper,^27^ the TacTip-GR2 combines the design features of the TacTip sensor with a reduced overall form factor (44 mm; Table 1). A smaller camera (Adafruit spy camera for Raspberry Pi) and fisheye lens replace the Microsoft LifeCam HD webcam, to enable this reduction in size.

**Table 1.** Details of Pin Properties for Each Sensor of the TacTip Family

*Sensor*	*Sensor dimensions (mm)*	*Number of pins*	*Pin dimensions (mm)*
TacTip	40 × 40 × 85	127	1.2 × 2.0
TacTip-GR2	40 × 40 × 44	127	1.2 × 2.3
TacTip-M2	32 × 102 × 95	80	1.5 × 2.1
TacCylinder	63 × 63 × 82	180	3.0 × 3.0

The pin layout of the modular tips is maintained for this version of the TacTip, but a flatter skin component creates more space between the gripper's fingers, allowing larger objects to be grasped by the gripper.

This flatter skin (Fig. 4) leads to a change in sensor dynamics, with a smaller volume tip reducing the pin deflections but increasing the contact surface area for flat objects.

#### TacTip-M2

**FIG. 6.**
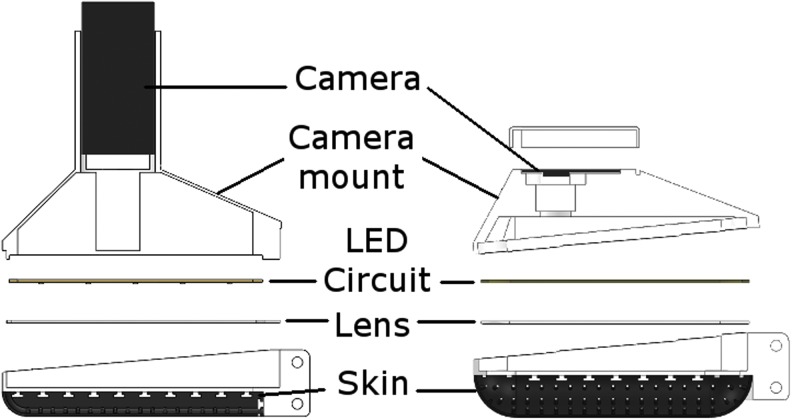
The original TacTip-M2 (*left*) is used when the form factor is nonessential and the improved TacTip-M2 (*right*), more compact, is designed for integration onto the OpenHand M2 gripper.

Applying TacTip design principles to the OpenHand model M2 gripper,^26^ we created the TacTip-M2,^10^ an elongated tactile thumb (Fig. 6) for application to in-hand dexterous manipulation of an object using only tactile feedback as guidance. We believe tactile manipulation to be an essential component in allowing robots to effectively interact with objects in complex, dynamic environments. The M2 gripper was chosen for integration as it is 3D printed and open source, has good grasping capability, and provides an opportunity for simple tactile manipulation to be investigated along one dimension.

As with the TacTip, the TacTip-M2 is fabricated through multimaterial 3D printing and has regularly spaced rows of pins on the inside surface of its skin. The TacTip-M2 features both an original version for deployment where form-factor is not an issue (e.g., for mounting at the end of a robot arm) and an improved, more compact version for integration on the M2 gripper featuring a rearranged webcam and macro lens.

#### TacCylinder

**FIG. 7.**
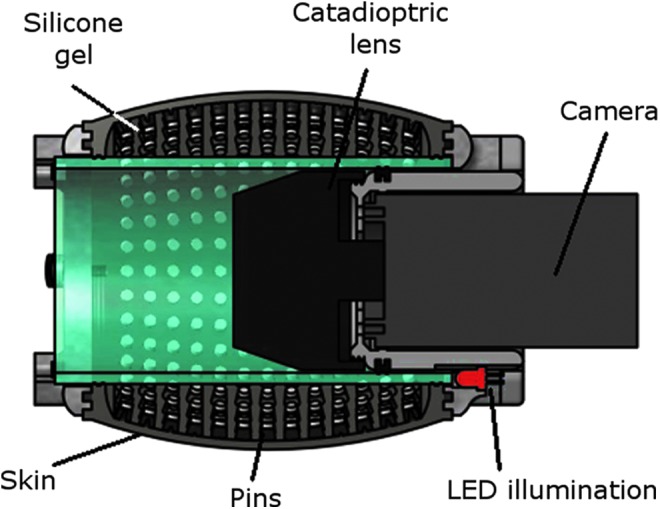
The TacCylinder is designed for capsule endoscopy. The cylindrical design comprises a 3D-printed cylindrical skin and catadioptric mirror system to achieve 360° tactile sensing.

The TacTip has been adapted with a catadioptric mirror system to provide a 360° cylindrical tactile sensing surface (Fig. 7), forming the TacCylinder sensor.

The TacCylinder is designed for capsule endoscopy, providing remote tactile sensing capabilities within the gastrointestinal tract.^28^ Capsule endoscopy is a pill-like technology swallowed by the patient, which travels through the intestines visually surveying the lumen for suspicious indications of ill health.

The TacCylinder is a larger sensor than the TacTip and thus contains more pins of larger dimensions (Table 1). A tube through its center holds the camera and a 360° mirror system. Filling the inside cavity of the sensor with the optically clear silicone gel is further aided by integrated 3D-printed O-ring-type seals.

### Experimental setup and data collection

**FIG. 8.**
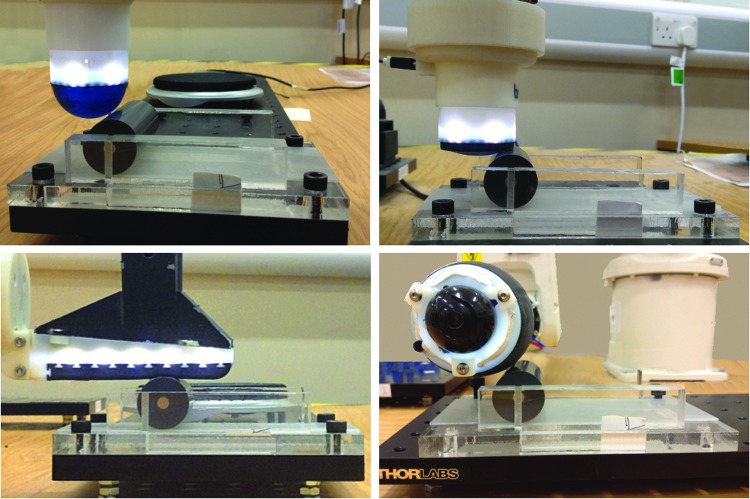
The TacTip, TacTip-GR2, TacTip-M2, and TacCylinder mounted on the ABB robot arm, with the 25 mm diameter cylinder being rolled over a 72 mm range.

For validation of perceptual performance, we test the TacTip, TacTip-GR2, TacTip-M2, and TacCylinder sensors on the same cylinder rolling task, to evaluate localization performance for each sensor. Note the TacTipGR2 is mounted on the standard TacTip body for convenience (Fig. 8). This experiment was chosen because it is simple to set up for all tactile sensors and was also compatible with the range of different designs, morphologies, and uses of the sensors, from manipulation tasks with robot hands to contacting objects with stand-alone sensors.

The sensors are attached as end-effectors to a 6-DOF ABB IRB120 industrial robot arm (Fig. 8), and brought into contact with a 25 mm diameter cylinder, by lowering the sensor's tip to 3 mm below its first point of contact with the cylinder. The cylinder is then horizontally rolled, with a custom platform constraining movements to one dimension (defined as *y*). The platform consists of a flat Perspex bottom plate, with two Perspex walls that constrain the cylinder to move along them (Fig. 8). A rubber surface is added to the bottom plate to ensure the cylinder does not slip while rolling. Magnets mounted at the ends of the cylinder and one end of the roller provide a home position for the cylinder.

The cylinder is rolled forward in a nonslip motion in 0.1 mm increments over a 72 mm range, totaling 720 different locations along the *y*-axis.

At each location, 10 images are recorded (640 × 480 pixels (px), sampled at ∼20 fps). These images are then filtered and thresholded in OpenCV (www.opencv.org), and the pin center coordinates are detected using a contour detection algorithm. Each pin is identified based on its proximity to a default set of pin positions recorded when the sensor is not in contact with the cylinder; if no pin is detected within a radius of 20 px from its default, the position from the previous frame is used instead. A time series of *x*- and *y*-deflections of the sensor's pins are then extracted and treated as individual taxel inputs. Several frames are collected to reduce noise arising from the pin detection algorithm and minor displacements of the sensor.

These data are collected twice for use as distinct training and test sets for offline cross-validation (see the Validation section), ensuring results are obtained from sampling on an independent set from the training data.

### Data processing

Formally, data are in the form of contact data ${z_{1:t}} = \left\{  {{z_1} , \; \ldots , \;{z_t}} \right\}$ encoded as a multidimensional time-series of sensor values

\begin{align*}
{z_t} = \left\{  {{s_k} \left( j \right) { \rm{ \;}}:1 \le j{ \rm{ \;}} \le {N_{{ \rm{samples}}}} , { \rm{ \;}}1 \le k{ \rm{ \;}} \le {N_{{ \rm{dims}}}}} \right\}  ,
\end{align*}

with indices $j , k \;$ labeling the time sample and data dimension, respectively. In our case, 10 frames are gathered per location, thus ${N_{{ \rm{samples}}}} = 10 \;$ and we consider *x*- and *y*-deflections of each of the pins as a separate dimension *k*, with ${N_{{ \rm{dims}}}} = 254$. These contact data give evidence for the present location class *y_l_*, $1 \le l \le {N_{{ \rm{loc}}}}$, considered one of a set of distinct punctual locations (here ${N_{{ \rm{loc}}}} = 72$ locations spanning 72 mm are used).

The location likelihoods ${P_k} ( {z_t} \vert {y_l} )$ use a measurement model of the training data for each location class *y_l_*.

\begin{align*}
\log P ( { z_t } \vert { y_l } ) = \mathop \sum \limits_ { k = 1 } ^ { { N_ { { \rm { dims } } } } } \mathop \sum \limits_ { j = 1 } ^ { { N_ { { \rm { samples } } } } } { \frac { \log { P_k } ( { s_k } \left( j \right) \vert { y_l } ) }  { { N_ { { \rm { samples } } } } { N_ { { \rm { dims } } } } } } 
\end{align*}

constructed by assuming all data dimensions *k* and samples ${s_k} \left( j \right)$ within each contact are independent (so individual log likelihoods sum). Here this sum is normalized by the total number of data points ${N_{{ \rm{samples}}}}{N_{{ \rm{dims}}}}$ to ensure that the likelihoods do not scale with the sample number of a contact.

As with previous work on robot tactile perception,^34,35^ the probabilities ${P_k} \left( {{s_k} \left( j \right) { \rm{ \vert }}{y_l}} \right)$ are found with a histogram method applied to training data for each location class *y_l_*. The sensor values *s_k_* for data dimension *k* are binned into equal intervals ${I_b} , \;1 \le b \le {N_{\rm bins}}$ over their range (here with ${N_{{ \rm{bins}}}} = 100$). The sampling distribution is given by the normalized histogram counts ${n_{kl}} \left( b \right)$ for training class *y_l_*:

\begin{align*}
 { { \rm { P } } _ { \rm { k } } } \left( { { { \rm { s } } _ { \rm { k } } } { \rm { \vert } } { { \rm { y } } _ { \rm { l } } } } \right) = { { \rm { P } } _ { \rm { k } } } \left( { { \rm { b \vert } } { { \rm { y } } _ { \rm { l } } } } \right) = { \frac { { n_ { kl } } \left( b \right) + \epsilon }  { \sum \nolimits_ { b = 1 } ^ { { N_ { { \rm { bins } } } } } { n_ { kl } } \left( b \right) } } , { \rm { \; } } 
\end{align*}

where ${n_{kl}} \left( b \right)$ is the sample count in bin *b* for dimension *k* over all training data in class *y_l_*.

Technically, the likelihood is ill-defined if any histogram bin is empty, which is fixed by regularizing the bin counts with a small constant ($\epsilon\gg 1$).

### Validation

Sensor validation provides an analysis of localization accuracy and algorithm performance using cross-validation performed after data collection. Two sets of data, termed training and testing, are gathered for cross-validation. Data *z_t_* is then sampled from the test set and classified according to a maximal likelihood approach, identifying the location *y_l_* based on the maximal location likelihoods $P ( {z_t} \vert {y_l} )$ of that contact data. The mean absolute error for each location class ${ \overline e _y} \left( y \right)$ is then evaluated over each test run at a given location, with the mean error ${ \overline e _y } = { \frac { \sum \nolimits_y { { \overline e } _y } \left( y \right) }  { { N_ { { \rm { loc } } } } } } \;$ the average over all locations.

## Results

### Inspection of data

#### TacTip

**FIG. 9.**
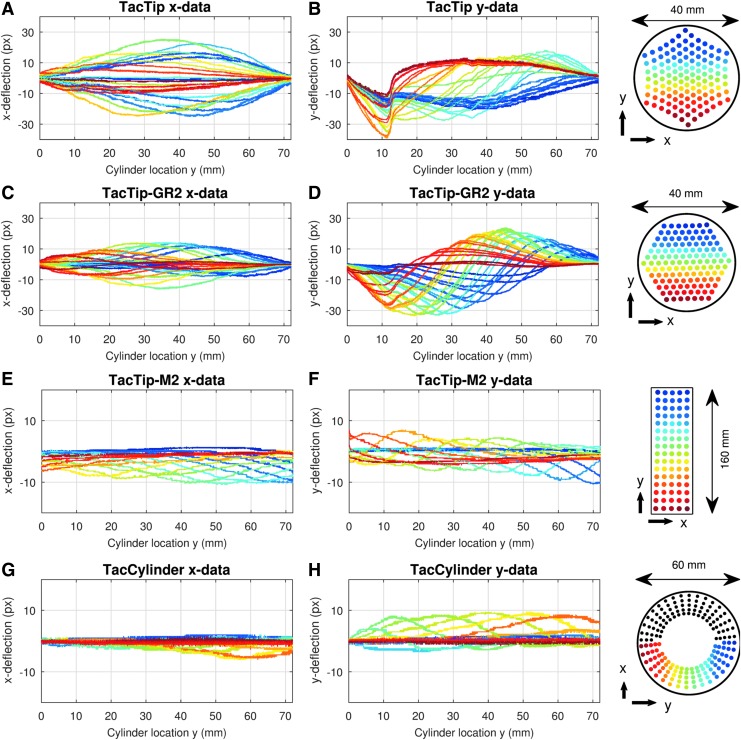
Tactile data from all four sensors. Data were sampled at 0.1 mm intervals along the 72 mm range of cylinder motion (720 samples). **(A, C, E, G)** pin displacements along the *x*-axis and **(B, D, F, H)** along the *y*-axis (direction of the cylinder roll). The four right-most panels identify pins for each sensor and display the *x*- and *y*-directions.

As the TacTip rolls the cylinder across a flat surface in the *y* direction (as described in the Experimental Setup and Data Collection section), we note the pin deflections in the *x*-direction (perpendicular to cylinder movement direction) have a regular pattern, with successive rows of pins deflecting outward (deflection reaching −30 to 30 px) and then returning to baseline (Fig. 9A, B). The *y*-deflections of pins (in the direction of cylinder motion) display an irregular pattern, however, with all pins initially dipping downward before recovering sequentially to baseline positions. This pattern is likely due to initial static friction between the cylinder and the sensor's skin.

#### TacTip-GR2

Data acquired from the TacTip-GR2 produced similar patterns of *x*- and *y*-deflections to the TacTip, although deflections are less pronounced (Fig. 9C, D). This is most visible in the *x*-direction, with an approximate deflection range of −12 to 12 px for the TacTip-GR2 (c.f. −30 to 30 px for the TacTip).

We note that the pins which contact the cylinder first (red and orange in Fig. 9) have the largest *y*-deflections in the TacTip case, whereas in the TacTip-GR2 data, the pins in the middle of the sensor (yellow and green in Fig. 9) are the most deflected in the *y* direction. This difference is a consequence of the shape of the sensors, with the TacTip's dome-shaped tip creating large *y*-deflections close to the initial contact. The TacTip-GR2 is mostly flat with a slight bulge around its center, creating a central area in which internal dynamics enables larger deflections. Note that these dome-shaped morphologies also explain the greater deflections of central pins (yellow and green) relative to pins around the sensors' edges (dark red, dark blue).

#### TacTip-M2

Data from the TacTip-M2 have a regular, repeated sinusoidal pattern, with a deflection range of −9 to 4 px in the *x*-direction and more pronounced deflections (−14 to 6 px) in the *y*-direction (Fig. 9E, F). This makes sense as it is the direction of movement of the cylinder, and is also the direction with most freedom of movement for pins, since it corresponds to the sensor's long axis. The sinusoidal pattern arises from the synchronized movement of rows of pins on the TacTip-M2 as the cylinder moves across them.

An asymmetry is also noticeable in both the *x*- and *y*-directions, with the magnitude of *x*-deflections increasing as the cylinder is rolled forward, and peaks of the sinusoidal pattern in the *y*-deflections gradually migrating downward from +8 to +2 px. This is likely due to the intrinsic mechanical asymmetry of the TacTip-M2, arising from the way in which the skin and base connect at each end of the sensor (Fig. 6).

#### TacCylinder

Data for the TacCylinder show a regular pattern of deflections (−6 to 2 px in the *x*-direction), which is greater in the *y*-direction (−4 to 12 px) (Fig. 9G, H). We only consider pins from the lower half of the TacCylinder, as the pins on the top half are unaffected by contact with the cylinder. We note a slight initial increase and then decrease of the peak amplitudes of deflections in the *y*-direction, showing that lower pins with more pressure applied are deflected further.

Thus, we can observe from the data gathered in this experiment from all four sensors that sensor morphology has a huge impact on the aspect and quality of collected data. The overall pattern of pin deflections, their relative and absolute amplitudes, and the order in which they deflect are all strongly dependent on sensor morphology. The next section explores how these differences affect performance on cylinder localization.

### Validation

#### TacTip

**FIG. 10.**
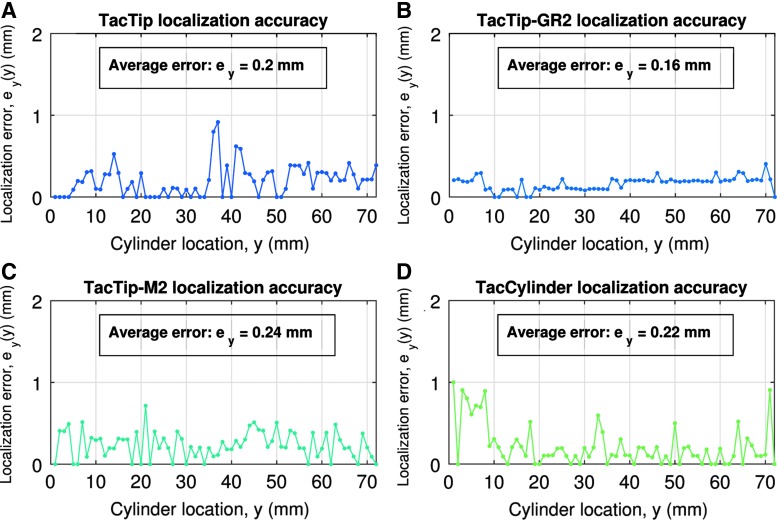
Localization accuracy of the four sensors: TacTip **(A)**, TacTip-GR2 **(B)**, TacTip-M2 **(C)**, and TacCylinder **(D)**. The results are shown for 72 location classes, each corresponding to a 1 mm range.

Localization performance of the TacTip is tested using the validation procedure detailed in the Materials and Methods (Validation section), and results are summarized in Table 2. The localization accuracy is an average ${ \overline e _y} = 0.20 \;{ \rm{mm}}$ over all location classes (Fig. 10A), and is below 1 mm everywhere. Considering the closest pins on the TacTip skin (in the center) are spaced 2.4 mm apart, and the *x*- and *y*-deflections of these pins act as taxels, we consider the resolution of the sensor to be 2.4 mm. As such, the TacTip demonstrates ∼12-fold super-resolution^7^ over the cylinder's movement range.

**Table 2.** Validation Results for Each Sensor from the TacTip Family

*Sensor*	${ \overline e _y}$*.* (mm)	*Pin spacing (mm)*	*Super-resolved accuracy*
TacTip	0.20	2.4	12-Fold
TacTip-GR2	0.16	2.4	15-Fold
TacTip-M2	0.24	3.5	15-Fold
TacCylinder	0.22	4.3	19-Fold

In a previous study,^31^ the TacTip with cast silicone skin was applied to the cylinder roll task as a demonstration of tactile manipulation along complex trajectories. That study found approximately eightfold super-resolved acuity; thus, our novel 3D-printed TacTip gives a similar order of super-resolved acuity.

#### TacTip-GR2

The TacTip-GR2's localization accuracy averages ${ \overline e _y} = 0.16 \;{ \rm{mm}}$ and remains below 0.3 mm over the entire range (Fig. 10B), corresponding to 15-fold super-resolution over the pin spacing of 2.4 mm. We interpret this slight improvement in localization relative to the TacTip sensor as a consequence of the flat surface of the TacTip-GR2 creating a more consistent pattern of pin deflections over the cylinder location range (Fig. 9).

#### TacTip-M2

Localization accuracy for the TacTip-M2 averages ${ \overline e _y} = 0.24 \;{ \rm{mm}}$ over all location classes (Fig. 10C), and submillimeter accuracy is evident over the full location range. Internal pins acting as taxels are spaced 3.5 mm apart on the sensor skin in the *x*- and *y*-directions. As such, the TacTip-M2 again demonstrates ∼15-fold super-resolution over the cylinder's movement range.

#### TacCylinder

Localization accuracy for the TacCylinder averages ${ \overline e _y} = 0.22 \;{ \rm{mm}}$ over all location classes (Fig. 10D), and submillimeter accuracy is displayed over most of the range of locations (3–72 mm). Note that the high errors on the initial range (0–7 mm) are linked to the TacCylinder not yet being fully in contact with the cylinder. Pins on the TacCylinder are spaced by a minimum of 4.3 mm on the sensor skin. As such, the TacCylinder demonstrates ∼19-fold super-resolution over the cylinder's movement range.

## Applications

### Manufacturing

The TacTip has been applied to a quality control task with potential applications to car manufacturing. In this study, active touch algorithms were used to identify gap widths to 0.4 mm accuracy, and vertical depth above the gap to 0.1 mm accuracy.^32^

Thus, mounting the TacTip on an industrial robot arm offers an accurate and reliable solution to automated quality control on the production line.

Another application of the TacTip sensor is in composite layup (Elkington et al. Using tactile sensors to detect defects during composite layup; unpublished data), in which tactile sensing could provide a real-time feedback to industrial robots to detect defects and irregularities during composite layup. This is a step toward fully automated composite layup, eliminating the need for costly and time-consuming manual “hand” layup.

The TacTip sensor thus presents solutions to the manufacturing industry to automate and potentially improve on tasks currently carried out manually.

### In-hand manipulation

**FIG. 11.**
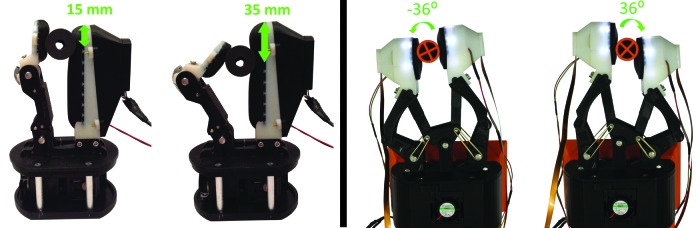
Range of movement for the M2 gripper (*left*) and the GR2 gripper (*right*) with integrated TacTips.

The TacTip-M2 is adapted for use on the M2 gripper.^26^ As such, objects can be rolled vertically up and down the sensorized gripper along a 20 mm range (Fig. 11, left panel). After training, trajectories can be followed based solely on tactile data, successfully performing one-dimensional in-hand tactile manipulation.^10^

The TacTip-GR2 integrated in the GR2 gripper can perform in-hand tactile reorientation of objects, a different form of tactile manipulation. Here both mobile fingers are tactile-enabled, rotating objects along a curved trajectory^36^ (Fig. 11, right panel).

Tactile manipulation tasks allow for the complex and precise handling of objects in-hand. These capabilities will enhance the safety, interactivity, and overall potential of robots in the fields of human–robot interaction, assistive and industrial robotics.

### Object exploration

Exploratory tactile servoing has been demonstrated with the TacTip in experiments involving several two-dimensional objects: a circular disk, a volute laminar, and circular or spiral ridges.^33^ A similar approach to that used here to validate the performance of the tactile sensors was used, adapted with principles from biomimetic active perception to perceive and control the edge orientation and radial location relative to the edge. The control policy rotated the sensor to maintain its orientation and radial location as the sensor moves tangentially along the edge, successfully following the contours of all the tested objects.

This approach combines active perception and haptic exploration into a common active touch algorithm, with the potential to generalize to more complex, 3D tasks. It also relates to human exploratory procedures^37^ (contour following here), and the control policy could thus be extended to include more of these exploratory procedures (for instance, enclosure to detect volume in a robot hand).

### Psychophysics

The TacTip was also used to investigate discrimination-based perception.^38^ In that study, the TacTip was trained to discriminate between two edges of different sharpnesses and obtained a just noticeable difference (JND) of 9.2°, comparing favorably to a previously reported human JND of 8.6°.

Future work with the TacTip sensors could further this approach to explore the concept of robo-psychophysics,^39^ in which human psychophysics experimental approaches are used to evaluate artificial sensors.

### Medical applications

Recent work by Winstone *et al.*^28^ has shown the ability of the TacCylinder to detect surface deformation of various lumps associated with suspect tissue that could reside within the gastrointestinal tract. These sensing data have been applied to create a 3D rendering of the test environment. Currently, work is focused on the discrimination between lump features and tissue density toward more accurate identification of submucosal tumors.^9^ Work has been carried out in parallel to create a self-contained pneumostatic palpating sensor.^40^

In the past, the TacTip^41^ has been used as part of a teletaction system for lump detection, in which tactile feedback is relayed to the surgeon. More recently, the TacTip's design principles were applied to a pillow used during magnetic resonance imaging (MRI) scans to detect subtle head movements.^42^ Thus, TacTip sensors hold promise for multiple medical applications, particularly in tumor detection, capsule endoscopy, and MRI scans.

### Further applications

Future iterations of TacTip sensors could create novel solutions for known practical problems in robotics, bringing tactile sensing to new areas and applications. The 3D-printed nature of these sensors and the open availability of CAD files and fabrication methods (softroboticstoolkit.com/tactip) enable easy use, adaptation, and improvement of the TacTip sensors. As well as further exploration of the areas described above, novel applications include patches of tactile skin to cover a robot surface or tactile feet to improve walking in bipedal robots.

## Discussion

We tested four 3D-printed sensors on a cylinder rolling task: the TacTip, TacTip-GR2, TacTip-M2, and TacCylinder. We found that all four sensors were able to localize the cylinder with submillimeter accuracy. All four sensors demonstrated above 10-fold super-resolution, with the TacTip-GR2 performing best (although it also had a closer pin spacing than the TacCylinder), possibly because its morphology is the most suited to a rolling task.

All the TacTip sensors utilize the same working principle, yet their different morphologies yield appreciable differences in behavior. These results reinforce the validity of the tight link between shape and function in a sensor and show the advantage of using 3D-printing techniques, which allow morphological customization. In particular, multimaterial printing enables a full sensor to be 3D printed, including its soft skin, opening up further possibilities of experimentation with different materials and morphologies. The sensor's compliance could be adjusted for different tasks by modifying the 3D-printed skin material and the shore hardness of silicone gel used in the tip. Further experiments on parameters such as pin length, pin spacing, and pin width could also reveal optimal solutions for TacTip designs applied to specific tasks. The TacTip-M2 and TacCylinder sensors could be made modular to facilitate these experiments. Directions for future work include accelerating the data processing algorithm and overall control loop to establish tasks with continuous, uninterrupted motion and miniaturizing the TacTip further for integration into a wide range of robotic hands.

To encourage the use of the TacTip design principles for new tactile sensing applications, we have open-sourced the hardware on the soft robotics toolkit (www.softroboticstoolkit.com/tactip) along with fabrication instructions. This submission won the “2016 contributions to soft robotics research” prize and aims to provide open access to cheap customizable tactile sensors, which are currently lacking from the field. It is our intention that research groups will use and develop TacTip sensors, and take advantage of 3D-printing technologies to apply our design principles to novel sensors and systems of their own devising.

## Conclusion

Soft tactile sensors are essential for manipulation tasks and safe human–robot interaction. Our suite of soft biomimetic tactile sensors displays strong super-resolved performance on localization tasks. These sensors provide a basis for future research and innovation in the field of tactile sensing.
